# Association between obesity and sleep disorder in the elderly: evidence from NHANES 2005–2018

**DOI:** 10.3389/fnut.2024.1401477

**Published:** 2024-08-29

**Authors:** Weifei Wang, Zhong Chen, Wenyuan Zhang, Rui Yuan, Yaqi Sun, Qi Yao, Jian Lu, Jungang Zheng

**Affiliations:** ^1^Department of Gerontology, The First Affiliated Hospital of Ningbo University, Zhejiang, China; ^2^Department of Anesthesiology, Beilun District People's Hospital, Ningbo, Zhejiang, China; ^3^Meigu County People's Hospital, Liangshan Prefecture, Sichuan, China; ^4^The Children's Hospital, Zhejiang University School of Medicine, National Clinical Research Center for Child Health, Hangzhou, China; ^5^Department of Anesthesiology, The First Affiliated Hospital of Ningbo University, Zhejiang, China; ^6^Department of Ultrasound in Medicine, The First Affiliated Hospital of Ningbo University, Ningbo, China; ^7^The First People's Hospital of Yuexi County, Liangshan Prefecture, Sichuan, China; ^8^School of Medicine, Zhejiang University, Hangzhou, Zhejiang, China

**Keywords:** body mass index, obesity, sleep disorder, NHANES, elderly

## Abstract

**Background:**

The available data exhibit inconsistent findings regarding the association between obesity and sleep problems among older adults. The objective of this study was to assess the potential association between obesity and sleep disorders in the older population.

**Methods:**

The data utilised in this cross-sectional investigation was obtained from the National Health and Nutritional Examination Survey (NHANES) conducted between 2005 and 2018. The study employed a multivariate logistic regression model and conducted subgroup analysis to evaluate the association between obesity and sleep disturbance.

**Results:**

The investigation consisted of 2,570 older people who provided complete information, out of which 324 individuals satisfied the criteria for sleep disturbance. The findings from the multivariable adjusted logistic regression model indicate that individuals in the overweight and normal weight groups exhibited decreased odds of experiencing sleep disorder, as evidenced by the adjusted odds ratios (AOR) of 0.46 (95% confidence interval [CI] = 0.34–0.61) and 0.33 (95% CI = 0.22–0.47), respectively. These results were statistically significant (*p* < 0.001) when compared to individuals in the obese group. The investigation of age and gender subgroups demonstrated similar associations between various BMI categories and sleep disorders in the older population.

**Conclusion:**

In summary, there exists a correlation between obesity and sleep disorders in the senior population. A significant association was observed between BMI and the likelihood of experiencing sleep disorders, indicating a dose–response relationship. Individuals with a higher BMI demonstrated a heightened likelihood of experiencing sleep disorders compared to those with a lower BMI.

## Introduction

Obesity and sleep disturbances are prominent health issues that have received considerable attention due to their adverse impacts on personal welfare and the broader public health landscape (1–3). The global incidence of these illnesses has been progressively rising, placing a significant strain on healthcare systems and requiring a thorough comprehension of their correlation within distinct populations (4, 5). The aged population is particularly vulnerable due to age-related physiological changes, which render them more prone to a range of health issues (6, 7).

In recent years, a considerable body of research has been dedicated to investigating the correlation between obesity and sleep disturbances, emphasising the reciprocal nature of this connection (8, 9). Obesity, which is defined as the excessive buildup of adipose tissue in the body, has been associated with a wide range of detrimental health consequences, such as cardiovascular disorders, type 2 diabetes, and specific forms of cancer (10, 11). Moreover, obesity is widely acknowledged as a substantial risk factor in the emergence of sleep disorders, including obstructive sleep apnoea (OSA), insomnia, and restless leg syndrome (RLS) (12, 13). Sleep disturbances can significantly impact the quality of life and general health of individuals, resulting in symptoms such as daytime weariness, cognitive impairment, and increased morbidity.

The aged demographic constitutes a distinctive subset of individuals with discernible physiological and behavioural attributes. The process of ageing is frequently linked to alterations in body composition, hormone patterns, and sleep structure, all of which may contribute to the onset of obesity and sleep disorders (14). Furthermore, it is important to note that the aged demographic is especially susceptible to the negative consequences associated with these illnesses as a result of preexisting comorbidities and decreased functional capacities. The comprehension of the correlation between obesity and sleep disturbances in the senior population holds significant significance in the context of public health planning and the development of intervention methods.

This study aims to investigate the correlation between obesity and sleep disturbances in the older population by utilising data obtained from the National Health and Nutrition Examination Survey (NHANES) conducted during the period of 2005 to 2018. The National Health and Nutrition Examination Survey (NHANES) is a survey conducted in the US that aims to gather complete health-related data. This nationally representative survey collects information on several aspects, such as demographics, medical conditions, and sleep patterns. Through the examination of this comprehensive dataset, our objective is to examine the frequency of obesity and sleep disorders within the older demographic, research the probable variables that contribute to their simultaneous occurrence, and evaluate the effects of these illnesses on the overall health outcomes of this susceptible population.

## Methods

### Study population

The National Health and Nutrition Examination study (NHANES) is a comprehensive and representative study conducted in the US. It gathers extensive data on the nutrition and health status of the general population. The survey data collected by NHANES are made publically accessible, allowing researchers and data users to access and analyse the information. The National Centre for Health Statistics (NCHS) collects its statistics in biennial cycles (15). In order to get substantial samples for analytical purposes, we aggregated seven cycles of the continuous National Health and Nutrition Examination Survey (NHANES) data spanning from 2005 to 2018. The survey protocol was approved by the NCHS Research Ethics Review Board, and all participants provided signed informed permission. Additional information regarding the National Health and Nutrition Examination Survey (NHANES) can be accessed through the official website of the Centres for Disease Control and Prevention (CDC).^1^

**Figure 1:**
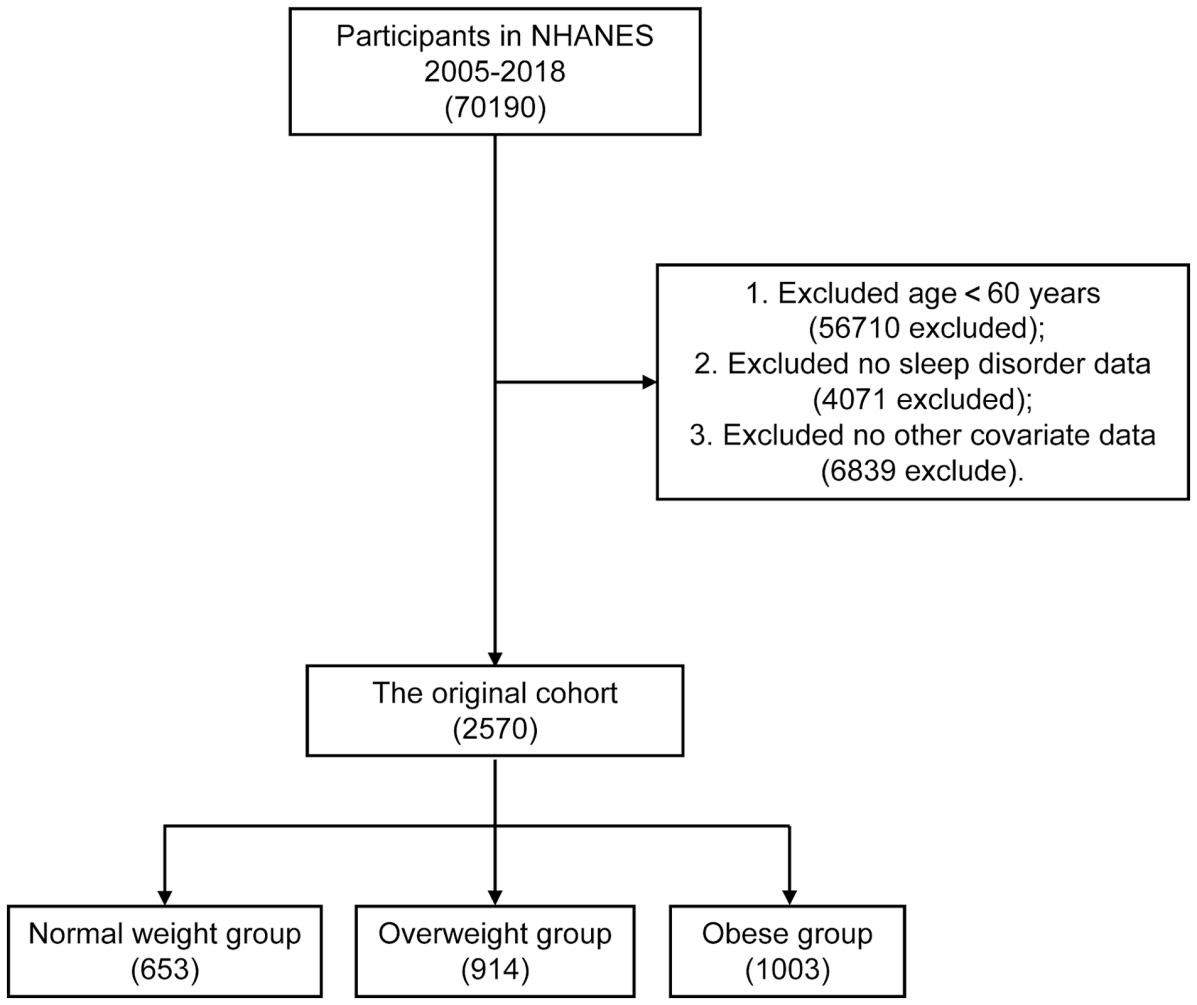
Screening of admissions for inclusion.

Sleep disorders are more common in older people. Therefore, it is of special clinical significance to identify the factors affecting sleep disorders in the elderly population (14, 16). Out of a total of 70,190 participants obtained from the NHANES database, we eliminated individuals who were younger than 60 years old (*n* = 56,710), those who had incomplete data on sleep problem (*n* = 4,071), and those who were lacking information on other variables (*n* = 6,839). Ultimately, a comprehensive total of 2,570 individuals were incorporated into the scope of this research endeavour. Figure 1 illustrates the flowchart depicting the systematic selection procedure.

### Exposure variates

Body mass index (BMI) was employed as a marker of obesity. BMI, serving as an indicator of total body fat, was determined by dividing the weight (in kilogrammes) by the square of the height (in metres). It was then categorised into four groups: underweight (< 18.5 kg/m^2^), normal weight (18.5–25 kg/m^2^), overweight (25–30 kg/m^2^), and obese (≥ 30 kg/m^2^) (17).

### Covariates

Covariates considered include several demographic characteristics: Age, gender, race, education level, marital status, depression, poverty income ratio (PIR), smoking status, alcohol intake, total daily calories and caffeine intake, and recreational physical activity. Education level was assessed by the question “What is the highest grade or level of school you have completed or the highest degree you have received?” [less than high school/high school graduate or above]. The participants were categorised into two groups based on their marital status: those who had a spouse and those who did not. The assessment of depressive symptoms was conducted utilising the 9-item Patient Health Questionnaire (PHQ-9) depression scale. This scale has nine questions that are derived from the symptoms of depression outlined in the Diagnostic and Statistical Manual of Mental Disorders, Fourth Edition (DSM-IV). The scoring system for each of the nine items ranges from 0 (not at all) to 3 (nearly every day). By summing the scores of these nine things, a total score within the range of 0 to 27 can be obtained (18). In the present investigation, those who obtained PHQ-9 total scores equal to or greater than 10 were classified as exhibiting clinically significant depression (CRD) (19). The study sample was stratified into four distinct groups based on their alcohol consumption patterns: non-drinker, 1 to <5 drinks/month, 5 to <10 drinks/month, or 10+ drinks/month. The participants were queried regarding their lifetime consumption of 100 cigarettes and their present smoking habits in order to ascertain the presence of both current and former smokers. The individuals included in the study were classified as former smokers if they were not now engaged in smoking behaviour, but had previously consumed a minimum of 100 cigarettes in their lifetime. In addition, we incorporated the variable of total daily caloric intake as a potential covariate. Medical professionals conducted diagnoses of various diseases, such as diabetes and stroke, by inquiring participants with the question, “Have you ever been told by a doctor or health professional that you have __?.” The individuals reported several comorbid conditions of interest, namely congestive heart failure, chronic obstructive lung disease (including emphysema and/or chronic bronchitis), coronary artery disease, malignancy, and hypertension.

### Outcome variable

The dependent variable in this study was the presence or absence of a sleep problem among the participants. An individual was classified as having a sleep disorder if they responded affirmatively to the query, “Have you been diagnosed with a sleep disorder?”

### Statistical analysis

Continuous variables are shown as medians [interquartile ranges (IQRs)]; categorical variables are presented as total numbers and percentages. Comparisons between groups were made using the χ^2^ test for categorical variables and the Mann–Whitney U test for continuous variables, as appropriate. We divided the blood lead concentration into four categories. The study employed multivariate logistic regression analysis to investigate the association between BMI and the presence of sleep disorders. Sleep disorder-related factors were identified by referencing existing literature and utilising data from the NHANES database. Subsequently, factors with a significance level of *p* < 0.05 in inter-group single factor analysis were recognised as confounders for inclusion in subsequent multivariate regression analysis. Model 1 in its initial form did not account for any potential confounding variables. In Model 2, adjustments were made for gender and age. In addition to the variables adjusted by Model 2, there are other potential confounding factors that need to be considered. To evaluate the potential nonlinear association of sleep disorder with BMI, the restricted cubic spline (RCS) analysis was performed in multivariable-adjusted models. RCS fits the predictor variable by segmenting it into a series of polynomial functions, with each segment being fitted using a low-order polynomial. The study conducted subgroup analysis, stratifying the data by age and gender. The statistical analyses were conducted using R software (version 4.1.1), and a significance level of *p* < 0.05 was employed to determine statistical significance.

## Results

### Description of the study population

This study comprised a sample of 2,570 adults ranging in age from 60 to 85 years who had available data on sleep disorders. Among the people included in the study, 49.5% (*n* = 1,272) fell within the age range of 60–69 years. This was followed by individuals aged 70–79 years, accounting for 28.0% (*n* = 720) of the sample. Lastly, those aged 80 years and above constituted 22.5% (*n* = 578) of the participants. The gender distribution exhibited a reasonably similar proportion. A subset of the study population, comprising 12.6%, consisted of individuals who reported experiencing sleep difficulties. There were significant differences seen in age, gender, obesity, depression, alcohol intake, diabetes, stroke, and comorbidity index between the sleep disorder group and the non-sleep disorder group (*p* < 0.05; Table 1). Individuals diagnosed with sleep disorders were more likely to be aged 60–69 years, male, have higher rates of obesity, increased prevalence of depression, a medical history that includes diabetes and stroke, and a higher comorbidity index.

**Table 1:** Baseline characteristics of subjects in the different groups.

Covariates	No sleep disorder group (*n* = 2,246)	Sleep disorder group (*n* = 324)	*p*
**Age**			0.045
60–69 years	1,094 (48.7%)	178 (54.9%)	
70–79 years	631 (28.1%)	89 (27.5%)	
80+ years	521 (23.2%)	57 (17.6%)	
**Sex**			0.006
Male	1,073 (47.8%)	182 (56.2%)	
Female	1,173 (52.2%)	142 (43.8%)	
**Race**			0.544
Mexican American	197 (8.8%)	26 (8.0%)	
Non-Hispanic Black	530 (23.6%)	71 (21.9%)	
Non-Hispanic White	1,095 (48.8%)	173 (53.4%)	
Other Hispanic	219 (9.8%)	31 (9.6%)	
Other/multiracial	205 (9.1%)	23 (7.1%)	
**Education**			0.280
Less than high school	1,102 (49.1%)	148 (45.7%)	
More than high school	1,144 (50.9%)	176 (54.3%)	
**BMI**			< 0.001
Normal weight	612 (27.2%)	41 (12.7%)	
Overweight	828 (36.9%)	86 (26.5%)	
Obese	806 (35.9%)	197 (60.8%)	
**Marital Status**			0.170
Have spouse	1,278 (56.9%)	198 (61.1%)	
Without spouse	968 (43.1%)	126 (38.9%)	
**Depression**	164 (7.3%)	64 (19.8%)	< 0.001
**PIR**	2.18 [1.20–4.16]	2.19 [1.16–4.06]	0.615
**Smoking status**			0.947
Never	2001 (89.1%)	288 (88.9%)	
Current smoker	228 (10.2%)	34 (10.5%)	
Former smoker	17 (0.8%)	2 (0.6%)	
**Alcohol intake**			0.044
Non-drinker	740 (32.9%)	101 (31.2%)	
1–5 drinks/month	1,048 (46.7%)	175 (54.0%)	
5–10 drinks/month	360 (16.0%)	39 (12.0%)	
10+ drinks/month	98 (4.4%)	9 (2.8%)	
**Total calories**	1720.0 [1329.1–2186.9]	1759.0 [1361.0–2166.5]	0.743
**Total caffeine**	100.50 [30.00–197.50]	98.00 [32.50–212.25]	0.388
**Recreational physical activity**	955 (42.5%)	129 (39.8%)	0.389
**Diabetes**	1754 (78.1%)	198 (61.1%)	< 0.001
**Stroke**	152 (6.8%)	40 (12.3%)	0.001
**Comorbidity index**			< 0.001
1	1,034 (46.0%)	132 (40.7%)	
2	624 (27.8%)	52 (16.0%)	
3 or greater	588 (26.2%)	140 (43.2%)	

### Baseline comparison of different BMI groups

Of the participants included in the study, 653 participants were between 18.5 and 25 kg/m^2^ (Normal weight group), 914 participants were between 25 and 30 kg/m^2^ (Overweight group), and 1,003 patients were ≥ 30 kg/m^2^ (Obese group). Table 1 lists the demographic features of all participants between different BMI groups. In different groups of BMI, age, gender, race, marital status, rates of depression, PIR, alcohol intake, smoking status, recreational physical activity, diabetes, comorbidity index, and rates of sleep disorder are significantly different (*p* < 0.05; Table 2).

**Table 2:** Baseline characteristics of subjects in the different BMI levels groups.

Covariates	Normal weight group (*n* = 653)	Overweight group (*n* = 914)	Obese group (*n* = 1,003)	*p*
**Age**				< 0.001
60–69 years	303 (46.4%)	427 (46.7%)	542 (54.0%)	
70–79 years	168 (25.7%)	262 (28.7%)	290 (28.9%)	
80+ years	182 (27.9%)	225 (24.6%)	171 (17.0%)	
**Sex**				< 0.001
Male	336 (51.5%)	399 (43.7%)	580 (57.8%)	
Female	317 (48.5%)	515 (56.3%)	423 (42.2%)	
**Race**				< 0.001
Mexican American	35 (5.4%)	83 (9.1%)	105 (10.5%)	
Non-Hispanic Black	132 (20.2%)	170 (18.6%)	299 (29.8%)	
Non-Hispanic White	325 (49.8%)	475 (52.0%)	468 (46.7%)	
Other Hispanic	49 (7.5%)	107 (11.7%)	94 (9.4%)	
Other/multiracial	112 (17.2%)	79 (8.6%)	37 (3.7%)	
**Education**				0.085
Less than high school	295 (45.2%)	446 (48.8%)	509 (50.7%)	
More than high school	358 (54.8%)	468 (51.2%)	494 (49.3%)	
**Marital Status**				0.002
Have spouse	366 (56.0%)	567 (62.0%)	543 (54.1%)	
Without spouse	287 (44.0%)	347 (38.0%)	460 (45.9%)	
**Depression**	43 (6.6%)	56 (6.1%)	129 (12.9%)	< 0.001
**PIR**	2.31 [1.22–4.35]	2.28 [1.23–4.30]	1.97 [1.14–3.87]	0.007
**Alcohol intake**				0.002
Non-drinker	199 (30.5%)	271 (29.6%)	371 (37.0%)	
1–5 drinks/month	281 (43.0%)	448 (49.0%)	494 (49.3%)	
5–10 drinks/month	142 (21.7%)	157 (17.2%)	100 (10.0%)	
10+ drinks/month	31 (4.7%)	38 (4.2%)	38 (3.8%)	
**Smoking status**				< 0.001
Never	546 (83.6%)	819 (89.6%)	924 (92.1%)	
Current smoker	102 (15.6%)	90 (9.8%)	70 (7.0%)	
Former smoker	5 (0.8%)	5 (0.5%)	9 (0.9%)	
**Total calories**	1732.5 [1347.0–2177.0]	1757.3 [1348.3–2227.0]	1699.5 [1280.5–2142.8]	0.050
**Total caffeine**	92.50 [26.000–192.50]	105.50 [31.625–207.63]	98.00 [29.00–197.25]	0.206
**Recreational physical activity**	313 (47.9%)	407 (44.5%)	364 (36.3%)	< 0.001
**Diabetes**	96 (14.7%)	183 (20.0%)	339 (33.8%)	< 0.001
**Stroke**	51 (7.8%)	58 (6.3%)	83 (8.3%)	0.257
**Comorbidity index**				< 0.001
1	267 (40.9%)	400 (43.8%)	499 (49.8%)	
2	235 (36.0%)	256 (28.0%)	185 (18.4%)	
3 or greater	151 (23.1%)	258 (28.2%)	319 (31.8%)	
**Sleep disorder**	41 (6.3%)	86 (9.4%)	197 (19.6%)	< 0.001

### Association of BMI with sleep disorder

The incidence of sleep disorder was significantly higher among patients with Obese group (19.6%; 197 of 1,003), compared with patients with Overweight group (9.4%; 86 of 914), and Normal weight group (6.3%; 41 of 653; *p* < 0.001; Table 2). We have used three multivariate logistic regression models to show the relationship between BMI with sleep disorder in Table 3: model 1, no covariate was adjusted; model 2, age, and gender were adjusted; model 3, age, gender, race, marital status, rates of depression, PIR, alcohol intake, smoking status, recreational physical activity, diabetes, comorbidity index, and rates of sleep disorder were adjusted. We found a significantly positive association between BMI with the incidence of sleep disorder in the unadjusted model (Model 1). In model 2, the ORs (95% CIs) after adjusting for age and gender for incidence of sleep disorder in participants with Overweight weight group and Normal group compared with those with Obese group were 0.40 (0.31–0.53), 0.27 (0.19–0.38), respectively (Table 3, *p* < 0.001). In model 3, the ORs (95% CIs) after adjusting for related indexes for incidence of sleep disorder in participants with Overweight weight group and Normal group compared with those with Obese group were 0.46 (0.34–0.61), 0.33 (0.22–0.47), respectively (Table 3, *p* < 0.001). For sensitivity analysis, different BMI levels group were transformed into a categorical variable, and the *p* value for the trend of different BMI levels group with categorical variables was consistent with the result of different BMI levels group as a continuous variable in the different models (Table 3, *P*_trend_ < 0.001).

**Table 3:** Logistic regression model of the effect of different levels of BMI on sleep disorder.

Groups	*β*	OR (95%CI)	*p*	*P* for trend
**Model 1** ^a^				<0.001
Obese		1.00		
Overweight	−0.856	0.42 (0.32–0.56)	<0.001	
Normal weight	−1.294	0.27 (0.19–0.39)	<0.001	
**Model 2** ^b^				<0.001
Obese		1.00		
Overweight	−0.907	0.40 (0.31–0.53)	<0.001	
Normal weight	−1.309	0.27 (0.19–0.38)	<0.001	
**Model 3** ^c^				<0.001
Obese		1.00		
Overweight	−0.780	0.46 (0.34–0.61)	<0.001	
Normal weight	−1.118	0.33 (0.22–0.47)	<0.001	

a Unadjusted.

b Adjusted for age, and sex.

c Adjusted for age, sex, race, marital status, depression, PIR, alcohol intake, smoking status, recreational physical activity, diabetes, and comorbidity index.

### The nonlinear analyses of the association between continuous BMI and sleep disorder

**Figure 2:**
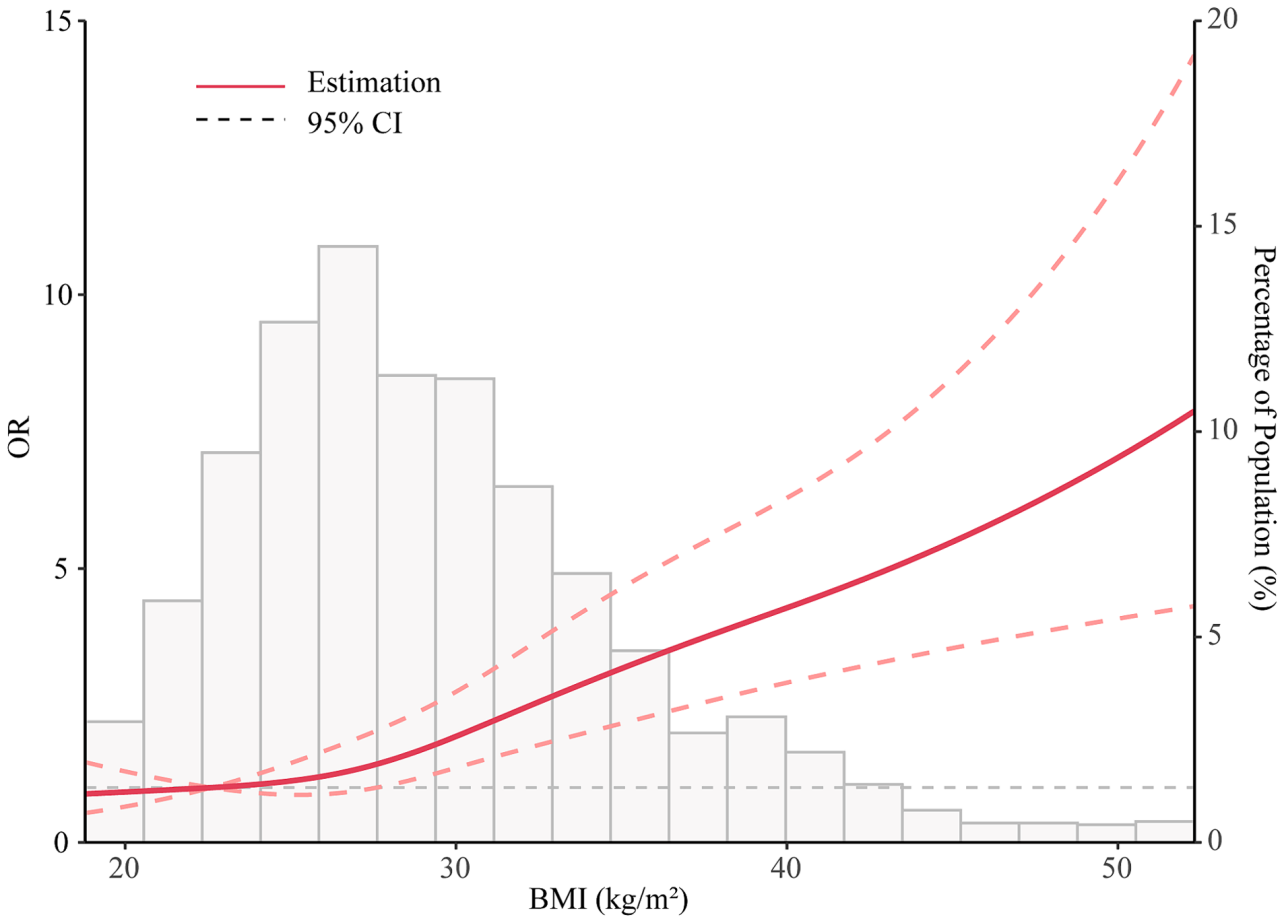
Cubic spline analyses of the associations of BMI with sleep disorder.

Spline models with fully adjusted covariates were constructed to profile a more direct relationship between BMI and sleep disorder. A J-shaped association was observed between BMI and sleep disorder (*p* < 0.001, and nonlinear *p* = 0.186; Figure 2).

### Subgroup analyses

**Figure 3:**
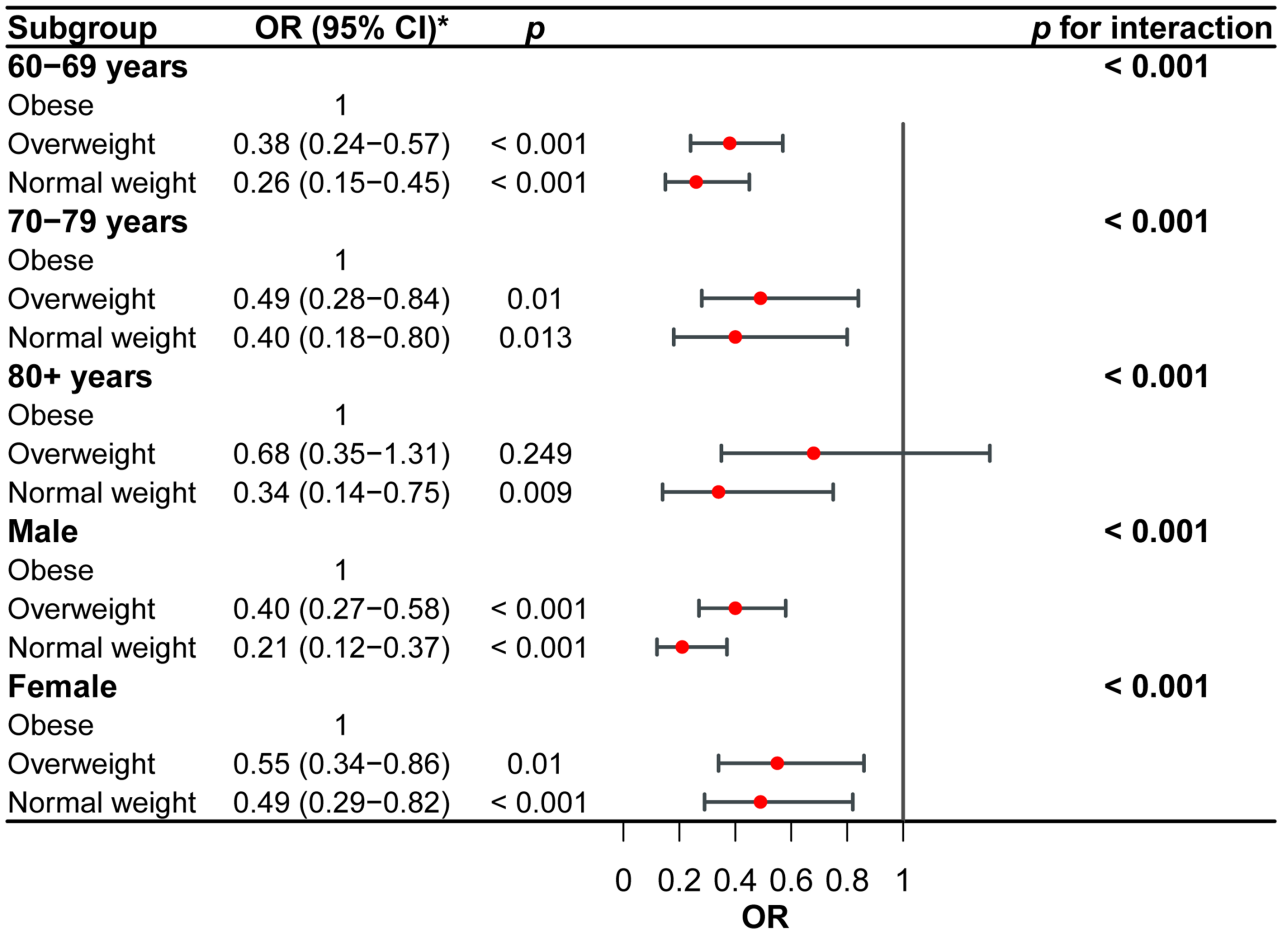
Subgroup analyses for the correlation between different BMI levels and incidence of sleep disorder. *Adjusted for age, sex, race, marital status, depression, PIR, alcohol intake, smoking status, recreational physical activity, diabetes, and comorbidity index.

Figure 3 presents subgroup analyses pertaining to the connection between various levels of BMI and the occurrence of sleep disorders. The individuals involved in the study were categorised into subgroups based on their age and gender. The findings of the study indicate a consistent and significant relationship between various BMI levels and the occurrence of sleep disorders across different subgroups (Figure 3, *P*_trend_ < 0.05).

## Discussion

The primary objective of this study was to examine the correlation between obesity and sleep disturbances among older individuals. The data utilised for this investigation was obtained from the NHANES conducted throughout the period of 2005 to 2018. The results of our study offer significant contributions to the understanding of the correlation between these two crucial health issues among the aged population. This research provides essential knowledge that can inform public health strategies and guide treatment approaches.

The existing data demonstrate inconclusive findings regarding the correlation between obesity and sleep disorders. A cross-sectional population-based study conducted in the U.S. revealed a significant association between short sleep duration and all categories of abnormal body weight, including underweight, overweight, and obesity (20). However, a longitudinal observation within a large national cohort of Thai adults did not find any significant association between short sleep duration and underweight (21). Another study involving longevity Chinese individuals found no association between BMI and sleep quality (22). These inconsistencies in findings may stem from variations in ethnicity, characteristics of the study population, and the presence of other confounding factors.

The principal finding of our study reveals a statistically significant positive correlation between obesity and sleep disturbances among the senior population. The study revealed a significant association between sleep problems and obesity, indicating that individuals with sleep disorders exhibit a considerably greater incidence of obesity as compared to those without such illnesses. The aforementioned observation aligns with prior studies conducted on individuals from both younger and older age groups, highlighting the significance of obesity as a contributing factor to the onset and worsening of sleep disruptions (23–26).

The precise mechanisms that connect BMI and sleep disorders remain incompletely comprehended; however, various viable hypotheses can be posited. There exists a correlation between the buildup of visceral fat in the abdominal region and an augmented secretion of inflammatory cytokines and adipokines. This physiological response has been found to have adverse effects on sleep patterns, leading to a deterioration in the overall quality of sleep (27–29). Metabolic abnormalities, such as insulin resistance, dyslipidemia, and inflammation, are commonly observed in individuals with central obesity. These abnormalities have been found to potentially disrupt the regular sleep–wake cycle and contribute to sleep disorders (30). Furthermore, one additional plausible mechanism that may explain the correlation between obesity and sleep disturbances is the existence of obstructive sleep apnoea (OSA). OSA is a prevalent sleep disease characterised by the partial or total obstruction of the upper airway during sleep. This obstruction results in recurrent interruptions in breathing and the disruption of sleep patterns. OSA exhibits a robust correlation with obesity, particularly among older individuals. This association is attributed to age-related alterations in the upper airway, which can intensify the collapse of the airway during sleep (31, 32). Furthermore, it is worth noting that inflammation associated with obesity and changes in adipose tissue could potentially play a role in the disturbance of sleep patterns and the emergence of various sleep disorders, including insomnia and restless leg syndrome (RLS) (33–35).

The utilisation of multivariate logistic regression analysis in our study enabled us to account for any confounding variables, hence enhancing the reliability of our evaluation of the association between obesity and sleep disturbances among the older population. The findings of the study revealed that older adults who were classified as overweight or normal weight had significantly reduced likelihood of suffering sleep disorders in comparison to persons classified as obese. The results of this study indicate the presence of a dose–response correlation, wherein greater BMI levels are linked to a heightened susceptibility to sleep disruptions. In our subgroup analysis, following adjustment for confounding factors, we observed consistent findings across various age and gender cohorts. Weight management therapies targeting obesity in the senior population may offer potential benefits in terms of enhancing sleep quality and mitigating the likelihood of sleep disturbances. Our study highlights the significance of addressing weight management in the senior population as a means to improve healthy sleep patterns and general health, given that obesity is a risk factor that can be modified. Lifestyle treatments, encompassing dietary and exercise programmes, customised to address the unique requirements and constraints of older individuals, have the potential to significantly alleviate the adverse effects of obesity on the quality of sleep.

However, we have to admit that our study has several shortcomings that need to be noted and addressed in further research: (1) NHANES is a well-representative survey, and in theory these results may be generalizable to the overall US population. However, there may be selection bias in the NHANES sampling process, which may result in a sample that is not fully representative of the entire US population; (2) Due to the cross-sectional design of the study, it is not possible to establish a causal relationship between obesity and sleep disturbances. There is a need for longitudinal studies to investigate the temporal association between these variables and to evaluate the potential of weight reduction therapies in enhancing sleep outcomes among older adults. (2) While we have made efforts to account for numerous covariates to mitigate confounding bias, sleep disorders are complex conditions influenced by various genetic and environmental factors. Consequently, there may be unidentified confounders not documented in the NHANES database that could contribute to the development of sleep disorders; (3) the utilisation of self-reported sleep problems may give rise to recall bias and subsequent misclassification. Self-reported measures are inherently subjective and may be influenced by various factors such as recall bias, individual perception of symptoms, and social desirability bias. Participants may underreport or overreport symptoms, leading to inaccuracies in the data. Despite these limitations, self-reported measures remain valuable tools in epidemiological research due to their practicality and cost-effectiveness. The integration of objective sleep measures, such as polysomnography, into future research endeavours has the potential to enhance the comprehensiveness and precision of evaluating sleep disruptions among older adults.

In summary, our research presents persuasive findings that substantiate the correlation between obesity and sleep disturbances among the senior demographic. The aforementioned discovery highlights the significance of treating obesity as a potential risk factor for sleep disturbances among the older population. It also emphasises the necessity for focused interventions that focus on promoting weight control and enhancing sleep health in this vulnerable demographic. Through the use of these measures, it is possible to augment the holistic welfare and standard of living of senior adults, hence potentially mitigating the impact of sleep-related problems on public health. Additional research and longterm studies are required to substantiate these findings and to go into the underlying processes of this association in more depth.

## Data Availability

Publicly available datasets were analysed in this study. This data can be found at: https://www.cdc.gov/nchs/nhanes/index.htm.

## Footnotes

^1^ http://www.cdc.gov/nhanes
